# Isoscape of Oxygen Stable Isotopes in Woods of the Amazon

**DOI:** 10.3390/molecules31091542

**Published:** 2026-05-06

**Authors:** Ana Claudia Gama Batista, Maria Gabriella da Silva Araújo, Isabela Maria Souza-Silva, Deoclécio Jardim Amorim, Fabiana Cristina Fracassi Adorno, Gabriela Bielefeld Nardoto, Vladimir Eliodoro Costa, Mario Tomazello-Filho, Niro Higuchi, Perseu da Silva Aparicio, Yasmin Lara Bezerra Vieira da Silva, Marta Silvana Volpato Sccoti, Ana Carolina Barbosa, Fabio José Viana Costa, João Paulo Sena-Souza, Gabriel J. Bowen, Luiz Antonio Martinelli

**Affiliations:** 1 Center for Nuclear Energy in Agriculture, University of São Paulo, Av. Centenario 303, Piracicaba 13416-000, Brazil; 2 Department of Ecology, Institute of Biological Sciences, University of Brasilia, Campus Universitário Darcy Ribeiro, Bloco E s/n, Brasília 70910-900, Brazil; 3 Stable Isotopes Center, Institute of Biosciences, Universidade Estadual Paulista (UNESP), Botucatu 18618-689, Brazil; 4 Department Forest Sciences, Luiz de Queiroz College of Agriculture, University of São Paulo, Av. Pádua Dias, 11, Piracicaba 13418-900, Brazil; 5 National Institute for Amazon Research, Av. André Araújo, 2936, Petrópolis, Manaus 69067-375, Brazil; 6 Forestry Engineering Department, State University of Amapá, Av. Pres. Vargas, 650-Central, Macapá 68901-258, Brazil; 7 Forestry Engineering Department, Federal University of Santa Maria, Av. Roraima n° 1000 Cidade Universitária Bairro-Camobi, Santa Maria 97105-900, Brazil; 8 Department of Forest Sciences, University of Lavras, P.O. Box 3037, Lavras 37203-202, Brazil; 9 National Institute of Criminalistic, Federal Police, Setor Policial Sul, Lote 7, Asa Sul, Brasília 70610-902, Brazil; 10 Department of Geosciences, State University of Montes Claros, Melo, Monte Carlos 39580-000, Brazil; 11 Geology and Geophysics Department, University of Utah, Salt Lake City, UT 84112, USA

**Keywords:** stable isotope, *δ*^18^O, isoscapes, random forest, multiple regression, Amazon, forensic tracking

## Abstract

Stable oxygen isotopes (*δ*^18^O) in wood provide integrative records of plant water use and regional hydroclimatic processes, offering a powerful framework for spatial ecological analysis in tropical forests. Here, we present the first regional-scale *δ*^18^O isoscapes for Amazonian wood based on 387 trees sampled across 25 sites. After α-cellulose extraction, *δ*^18^O values were modeled using multiple linear regression (MLR) and Random Forest (RF) approaches. A Moran’s I test revealed no significant spatial autocorrelation (*p* = 0.73), indicating that geostatistical interpolation methods such as kriging were not appropriate for this dataset. The MLR model based on site-average data achieved an R^2^ of 0.70, with a mean absolute error (MAE) of 0.56‰ and root mean square error (RMSE) of 0.68‰. The RF model showed comparable performance (R^2^ = 0.67; MAE = 0.64‰; RMSE = 0.77‰). Both approaches reproduced a coherent southeast-to-northwest gradient, with lower *δ*^18^O values in the western Amazon and higher values in the east, consistent with regional patterns in precipitation isotopic composition and evapotranspiration. These findings demonstrate that climate-driven statistical modeling effectively captures large-scale isotopic structure across the Amazon basin, providing a robust spatial representation of *δ*^18^O variability in tropical forest wood.

## 1. Introduction

Stable isotopes have been extensively utilized in both ecological and forensic sciences [1,2]. The isotopic composition of elements such as carbon, hydrogen, nitrogen, oxygen, and sulfur varies based on the climatic, geographic, and ecological characteristics of the environment [3]. These spatial variations, referred to as isoscapes, can be modeled using statistical and machine learning techniques such as kriging and Random Forest [4,5,6]. Isoscapes have proven valuable in tracing the geographic origin of materials ranging from timber and wildlife to human remains and narcotics [7,8,9,10].

These environmental signals are absorbed by plants and incorporated into organic tissues through well-established physiological fractionation processes [11,12]. In wood, cellulose is preferentially analyzed because it preserves the isotopic signal formed during biosynthesis and provides a more consistent representation of environmental conditions than bulk wood. Among stable isotopes, *δ*^18^O in tree-ring cellulose is particularly informative because it integrates both the isotopic composition of source water and leaf-level evaporative enrichment during transpiration. Consequently, *δ*^18^O values in cellulose provide a robust proxy for local hydroclimatic conditions and, ultimately, geographic origin [13].

Although isotopic spatial modeling is well established, important uncertainties remain regarding how different statistical approaches represent isotopic variability, particularly in complex tropical systems. Linear models, such as multiple linear regression (MLR), assume proportional relationships between predictors and response variables, whereas non-linear approaches, such as Random Forest (RF), can capture more complex interactions. However, the extent to which these methodological differences influence the resulting isoscapes remains poorly understood, especially in the Brazilian Amazon, where applications are urgently needed. In this context, improving model reliability is critical for applications such as forensic timber tracking, where accurate geographic attribution is essential. Recent studies have demonstrated the potential of isotopic models for this purpose [14,15].

In this study, we develop a regional *δ*^18^O isoscape for Amazonian tree-ring cellulose and explicitly compare the performance of MLR and RF models. We hypothesize that *δ*^18^O patterns are primarily driven by large-scale hydroclimatic controls, and that both approaches capture these gradients, but differ in predictive range and spatial representation. By evaluating these differences, we aim to clarify how modeling choices influence isotopic spatial patterns and to improve the methodological basis for isotope-based provenance studies in tropical forests.

## 2. Results

### 2.1. Multiple Linear Regression

The model with the lowest AIC included the following climatic variables: mat, vpd, vap, rh, and pet. The coefficients produced by this model are shown in Table 1. The model built on the site-average data achieved an R^2^ of 0.70, indicating that these predictors account for a significant portion of the variance in *δ*^18^O of the cellulose. The MAE was 0.56‰, and the RMSE was 0.68‰ (Table 2). The residuals followed a normal distribution, with no discernible pattern between the predicted values and the residuals. For the entire dataset, there was a decrease in the R^2^ to 0.42, and an increase in the MAE and RMSE to 0.92‰ and 1.10‰, respectively (Table 2).

The isoscape was generated over a regular spatial grid encompassing longitudes from −74.00 to −43.50 and latitudes from −16.67 to 4.50. The grid comprised 12.392 nodes, arranged in 183 longitudinal by 127 latitudinal divisions, with an approximate spatial resolution of 0.16° (equivalent to ~18 km^2^ per cell). Model predictions based on site-average data ranged from 22.7‰ to 35.0‰, with a median value of 26.1‰ and an interquartile range from 25.1‰ to 27.7‰. Predictions using individual tree *δ*^18^O values yielded a similar distribution, ranging from 23.8‰ to 33.2‰, with the same median (26.1‰) and interquartile range (25.1‰ to 27.7‰). These predicted values align closely with the observed *δ*^18^O measurements, which had a median of 25.8‰, interquartile range (24.8‰ to 26.7‰), and ranged from 22.1‰ to 30.0‰.

**Figure 1:**
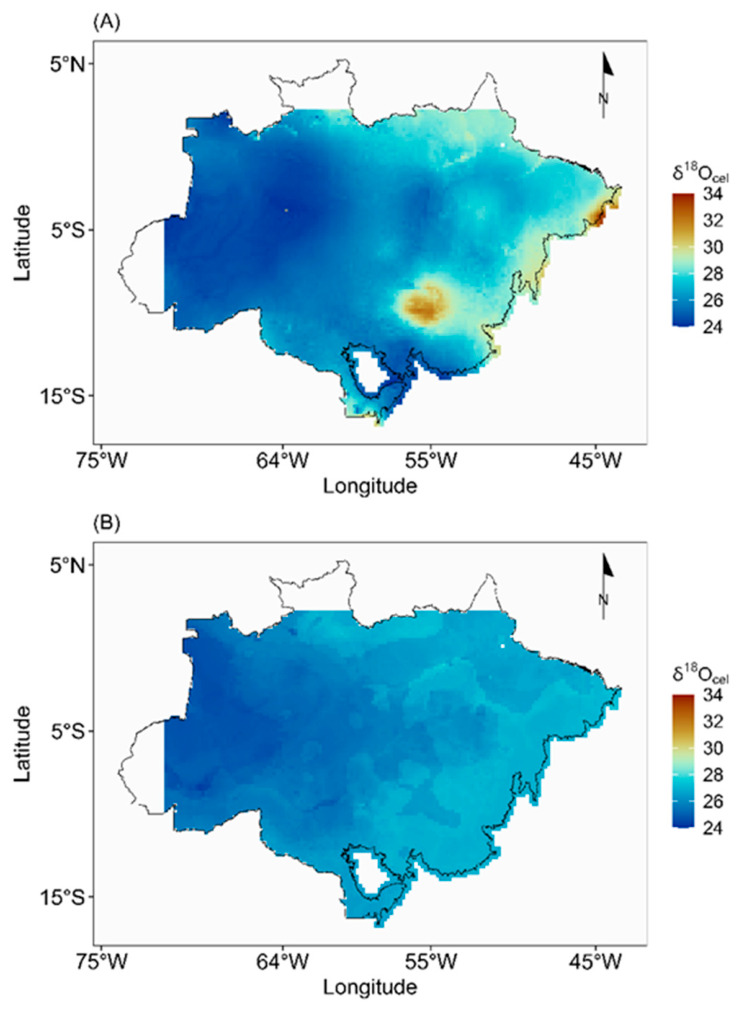
Average isoscapes of *δ*^18^O in cellulose across the Amazon region, modeled using (**A**) multiple linear regression (MLR) and (**B**) Random Forest (RF), based on site-averaged *δ*^18^O values. Black lines indicate the boundaries of the Amazon region. Warmer colors represent higher *δ*^18^O values, while cooler colors indicate lower values.

A marked spatial gradient in *δ*^18^O values is evident across the Amazon region, with values decreasing from east to west, particularly along a southeast-to-northwest axis (Figure 1A). Although Figure 1 presents the isoscape generated from site-average *δ*^18^O values, a remarkably similar spatial pattern was observed in the isoscape derived from individual tree-level data (Figure S2), reinforcing the robustness of the observed isotopic gradient across the region.

The standard deviation of the predictions using site-average *δ*^18^O values ranges from 0.72‰ to 1.9‰. Descriptive statistics indicate an interquartile range of 0.75‰ to 0.83‰, with a median of 0.78‰ and a mean of 0.83 ‰. The standard deviation of the predictions using individual trees *δ*^18^O values was higher, as expected. The median was 1.17‰, the interquartile range was from 1.16‰ to 1.19‰, and the range was from 1.15‰ to 2.05‰. Areas of low prediction uncertainty were distributed across the area, indicating relatively high confidence across much of the Amazon region (Figures S3 and S4). However, the areas with higher standard deviations tend to coincide with regions where sampling density is lower (Figure 1). Notably, many of these higher-uncertainty areas fall within the so-called “arc of deforestation”, a zone of intense land-use changes and ongoing illegal logging activity.

### 2.2. Random Forest

The Random Forest model trained on the site-average data, yielding the lowest MAE of 0.64‰, and RMSE of 0.77‰ and a coefficient of determination (R^2^) of 0.67, indicating that the model explains approximately 67% of the variance in the data (Table 2). Thus, such quality metrics are comparable with those produced by the multiple linear regression model. These metrics deteriorate when the whole dataset is used to train the model. The MAE and RMSE increased to 0.90‰ and 1.12‰, with a R^2^ of 0.44 (Table 2). Likewise, these metrics were also equivalent to those produced by the multiple linear regression model when using individual tree data.

Both the site-average and individual-tree isoscapes produced by the RF model showed similar spatial patterns, characterized by a southeast-to-northwest gradient in *δ*^18^O values. The site-average RF isoscape is presented in Figure 1B, while the individual-tree RF isoscape appears separately in the Supplementary Material (Figure S3). Despite these similarities, an important distinction emerged when comparing the RF and MLR model outputs: the range of predicted values was considerably narrower in the RF isoscape than in the MLR-based one (Figure 1A). Notably, the greatest spatial differences between the two models were found in the eastern Amazon, in the so-called “arc of deforestation”, where the models diverged most markedly (Figure 1B).

The standard deviation of the predictions using QRF and the site average dataset had slightly higher values than the standard deviations estimated using the MLR model. The median value was 0.87‰, the Q1 and Q3 were 0.70‰ and 1.10‰, respectively, and the range was from 0.2‰ to 2.0‰. However, the spatial distribution of standard deviation values across the Amazon was different than the one estimated by the MLR models. While the standard deviation isoscape by MLR produced a smooth surface with a gradual transition (Figures S3 and S4), the standard deviation isoscape by QRF produced patchy surfaces due to the differences in model structure between the two methods (Figures S5 and S6). The standard deviation isoscape produced by the QRF model exhibits a patchy spatial pattern due to its data-driven estimation of local prediction uncertainty, while the smoother standard deviation surface from the MLR model reflects its assumption of homoscedasticity and linear relationships between predictors and response.

## 3. Discussion

### 3.1. Comparison Between MLR and RF Models

This study represents the first effort to construct a *δ*^18^O isoscape from tree-ring cellulose across the Brazilian Amazon, with the broader aim of contributing to a multi-isotopic framework for tracing timber origin and helping combat illegal logging in the region. To evaluate how best to represent the spatial distribution of cellulose *δ*^18^O, we compared two modeling approaches, multiple linear regression (MLR) and Random Forest (RF), and two ways of structuring the data, using either individual-tree values or site-averaged values.

Our results show that MLR performed slightly better than RF, but the difference in predictive performance was small (Table 2). Cross-validation yielded comparable RMSE and R^2^ values for the two approaches, indicating that the main spatial variation in cellulose *δ*^18^O can be captured reasonably well by broad climatic gradients represented in a linear framework. This does not mean that nonlinear controls are absent, but rather that, at the spatial scale and sampling density of the present study, the additional flexibility of RF did not translate into a substantial gain in predictive accuracy.

The comparison also highlights an important distinction between models built from site-averaged values and those built from individual trees. Site-averaged models emphasize regional isotopic structure by reducing within-site variability and are therefore useful for describing broad geographic patterns. In contrast, models based on individual samples explicitly retain within-site variation and are more consistent with forensic provenance applications, where the unknown sample is a single tree rather than a site mean. The choice between these approaches therefore affects not only model performance, but also the practical interpretation of the resulting isoscapes.

**Figure 2:**
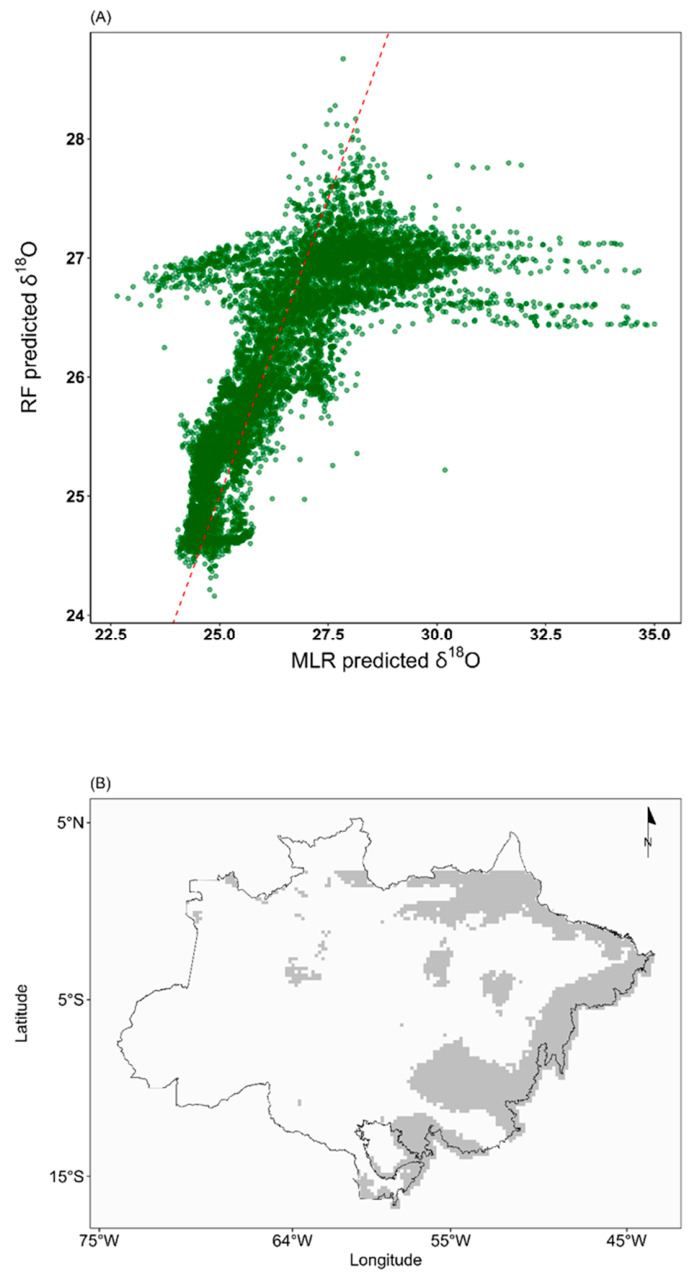
Comparison of *δ*^18^O predictions in tree-ring cellulose across the Amazon region from the multiple linear regression (MLR) and Random Forest (RF) models. (**A**) Scatterplot of *δ*^18^O predictions from both models. The dashed red line represents the 1:1 relationship. (**B**) Spatial map highlighting areas where the difference between MLR and RF predictions falls within the ±1‰ interval (white), and areas with differences exceeding ±1‰ (gray).

Although MLR and RF produced similar large-scale spatial patterns, the RF model predicted a narrower range of *δ*^18^O values than the MLR model (Figure 2). This likely reflects the tendency of RF to make conservative predictions within the range represented by the calibration data, whereas MLR can extend predictions more strongly along environmental gradients. In our case, the narrower RF range did not improve validation statistics, suggesting that the increased complexity of the RF approach did not provide a clear advantage for representing the basin-scale *δ*^18^O signal. At the same time, the broader range predicted by MLR should be interpreted cautiously until additional sampling, especially in underrepresented regions, allows these gradients to be tested more rigorously.

### 3.2. Spatial Distribution of Cellulose δ^18^O and Implications for Provenance

Both modeling approaches revealed similar broad spatial patterns in cellulose *δ*^18^O across the Brazilian Amazon, with lower values in the western basin, higher values toward the east, and intermediate values in central Amazonia. A latitudinal trend was also apparent, with relatively higher values toward the northern and southern margins and lower values in the central portion of the basin. The fact that these patterns were recovered by both MLR and RF suggests that they reflect real large-scale environmental gradients rather than artifacts of a particular modeling approach.

The east-to-west decrease in cellulose *δ*^18^O mirrors the isotopic gradient observed in rainfall across the Amazon Basin [6]. This pattern is consistent with Rayleigh-type fractionation during inland transport of Atlantic-derived moisture, in which successive condensation and precipitation events progressively remove the heavier isotope from atmospheric vapor [16,17]. As air masses move westward across the basin, the remaining vapor becomes increasingly depleted in ^18^O, and this signal is ultimately recorded in plant cellulose. Higher *δ*^18^O values in the eastern Amazon are therefore consistent with shorter transport distances and less cumulative rainout, whereas lower values in the west reflect stronger isotopic distillation and greater continental recycling of moisture. The agreement between rainfall isotope patterns and the cellulose isoscape indicates that tree-ring *δ*^18^O captures large-scale hydroclimatic structure across Amazonia.

Most predicted standard deviations in the isoscapes were below 1‰, but some localized areas showed substantially higher uncertainty, particularly in the standard deviation surfaces derived from the MLR model (Figures S2 and S3). Many of these areas overlap with the arc of deforestation, where sampling remains sparse and environmental disturbance is intense. This overlap is important because it identifies regions where provenance tools may be especially needed, but where the present isoscape is still less constrained. Future sampling and model refinement should therefore prioritize these zones in order to reduce uncertainty and improve the reliability of provenance assessments.

Considerable variation in *δ*^18^O was observed among individual trees within the same plots, likely reflecting species-specific physiological differences as well as local microenvironmental effects [18,19]. This within-site variability is highly relevant for provenance applications because timber tracing ultimately deals with individual trees rather than site means. It also reinforces the importance of extensive within-site sampling, since inadequate replication may underestimate the isotopic spread present at a given location and lead to overconfident geographic assignments.

The usefulness of the *δ*^18^O isoscape for timber provenance ultimately depends on the balance between spatial isotopic contrast and model uncertainty. Across the basin, the predicted range in *δ*^18^O is approximately 3‰, whereas model error is about 0.7‰. This means that geographic differentiation is most robust where isotopic gradients are strong, such as between the far western and far eastern Amazon, but becomes much less certain in low-contrast areas of central and eastern Amazonia. The present isoscape therefore has clear potential for broad regional discrimination, but limited power for fine-scale geographic assignment when used alone. Improving this resolution will require denser sampling and, most likely, integration with complementary tracers such as *δ*^2^H and ^87^Sr/^86^Sr, particularly in regions where isotopic homogeneity coincides with intense logging pressure.

## 4. Materials and Methods

### 4.1. Isotopic Data Collection

**Figure 3:**
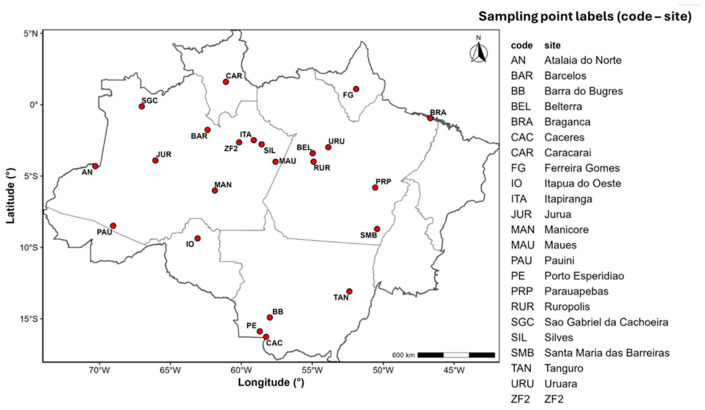
Map of sample distribution in the Amazon basin. The red dots indicate the sampling locations, covering various regions of the Legal Amazon, including National Forest (Flonas) and strategic areas for isotopic wood analysis.

To construct the *δ*^18^O-isoscape, we sampled 387 trees across 25 locations throughout the Amazon Basin (Figure 3). Species of commercial importance were initially targeted. However, due to the challenges of sampling the same species across the Amazon, we expanded the sampling strategy to include mature trees regardless of their commercial significance.

Representatives from 58 genera distributed across 24 botanical families were collected. These forest species represent different ecological groups, including pioneers, secondary, and climax or later-successional, occurring in mixed or dense ombrophilous forests and reflecting the phytosociology diversity of the Amazon region (Table S1).

Samples were collected from the base of trees harvested under authorized forest management plans within the Amazon region, in accordance with National Council for the Environment (CONAMA) Resolution N° 406 of 2 February 2009 [20]. The minimum cutting diameter (MCD) was ≥50 cm, and discs with a minimum thickness of 5 cm were obtained. Each wood disc was processed radially from the pith to the bark, and 2 mm thick laths were prepared, with their length varying according to the diameter of the collected disc.

**Figure 4:**
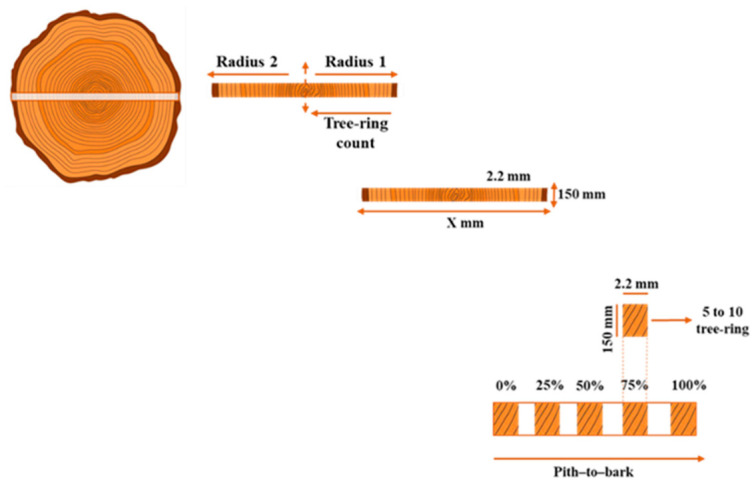
Preparation of the tree-ring laths. An entire radial section from pith to bark was removed from both sides of the stem disc. As both sides were considered symmetrical, the radius was divided in half, and ring counting started at the pith. The length of the laths was not standardized and depended on the disc diameter; however, all laths were sawn to a uniform thickness of 2 mm. The laths were marked at 0% (pith), 25% and 50% (heartwood), 75% (heartwood–sapwood transition), and 100% (sapwood–bark transition). These segment positions were defined to maximize the number of growth rings per section. The segments were cut into 2 cm pieces, each containing approximately 5–10 growth rings.

The laths were proportionally segmented along the radial at (0% (pith), 25%, and 50% (heartwood), 75% (heartwood–sapwood transition), 100% (sapwood up to the bark) (Figure 4). Each segment has approximately 5 to 10 growth rings, enabling characterization of the radial variation in isotopic signal.

The tree-ring laths were chemically treated to obtain α-cellulose following the protocols established by the Isotopic Ecology Laboratory—CENA/USP, which are based on previously published and widely applied methodologies [21,22]. Isotopic analyses were partially conducted at the Stable Isotope Laboratory of the University of California, Davis (USA), and at the Stable Isotope Center at São Paulo State University (UNESP), Botucatu, Brazil. An intercalibration procedure was performed between laboratories to ensure analytical consistency and standardization of the results.

Oxygen isotope analyses (^18^O/^16^O) were performed using continuous-flow isotope ratio mass spectrometry (CF-IRMS; Delta V Advantage, Thermo Fisher Scientific, Bremen, Germany) with an isotope ratio mass spectrometer (Delta V Advantage, Thermo Fisher Scientific, Bremen, Germany) coupled to a high-temperature conversion elemental analyzer (Flash HT elemental analyzer (EA; Thermo Fisher Scientific, Milan, Italy) via a gas interface (ConFlo IV, Thermo Fisher Scientific, Bremen, Germany).

The isotopic composition is reported in delta notation (*δ*^18^O), expressed in per mil (‰) relative to the Vienna Standard Mean Ocean Water (VSMOW) [23], following the recommendations of the International Union of Pure and Applied Chemistry (IUPAC) [24], according to Equation (1):

(1) $$\delta =\left(\frac{R_{sample}}{R_{standard}}-1\right)\times \;1000\;$$

where R represents the ^18^O/^16^O ratio of the sample and the international reference standard (VSMOW).

Calibration and quality control were performed using internationally recognized USGS reference materials (USGS54, USGS55, USGS56, USGS90, and USGS91). The certified *δ*^18^O values of these standards and their associated uncertainties were used to normalize the analytical data. A two-point normalization approach was applied to correct for instrumental drift and scale compression. Analytical precision, based on repeated measurements of reference materials, was better than ±0.26‰ for *δ*^18^O.

### 4.2. Environmental Data

Climatic predictors, including mean annual temperature (MAT), mean annual precipitation (MAP), potential evapotranspiration (PET), and vapor pressure deficit (VPD) were sourced from the WorldClim database (version 2.1; 2020 release). These variables were provided as monthly raster layers with a spatial resolution of 10 arc-minutes (~340 km^2^). Each variable is distributed as a set of 12 GeoTIFF files representing monthly conditions from January to December. All raster processing and data extraction procedures were carried out in QGIS- QGIS Geographic Information System (version 3.34; QGIS Development Team, Open Source Geospatial Foundation, Beaverton, OR, USA) [25].

Topographic information was incorporated using the Digital Elevation Model (DEM) available within WorldClim, which is derived from Shuttle Radar Topographic Mission (SRTM) data. Elevation layers share the same spatial resolution (10 arc-minutes) and have an approximate vertical accuracy of 10 m. These data were processed consistently with the climatic variables.

Atmospheric pressure (PA) was estimated from elevation using the standard barometric formulation (Equation (2)), which describes the decrease in pressure with increasing altitude:

(2) $$p(h)\;=P_{0}\;\;\times \;{\left(1-\frac{h}{44330}\right)}^{5.255}$$

where P(h) corresponds to atmospheric pressure at elevation h, P_0_ is the standard pressure at sea level (1013.25 hPa), and h represents elevation (m) derived from the DEM.

Relative humidity (RH) was derived from vapor pressure data. Raster layers of actual vapor pressure (kPa) were first obtained from WorldClim at the same spatial resolution. RH was then calculated as:

(3) $$RH\;=\;\frac{C_{a}}{\;e_{s}(T)}\;\times \;100$$

where RH is expressed as a percentage, C_a_ denotes actual vapor pressure, and e_s_(T) represents saturation vapor pressure as a function of temperature. The latter was computer following:

(4) $$e_{s}(T)=0.6108\;\times \;exp\;(\frac{17.24\;\times \;T}{237.3\;+\;T})$$

where e_s_(T) is given is kPa and T is air temperature (°C).

The isotopic composition of precipitation (δ^18^O_ppt_) was estimated using observations from the Global Network of Isotopes in Precipitation (GNIP). Spatial prediction was performed with the IsoriX package in R, which applies generalized linear mixed models (GLMMs) to generate continuous isotope surfaces (isoscapes). This framework integrates environmental predictors and spatial structure to estimate annual mean δ^18^O values across the region, allowing extraction of site-specific δ^18^O_ppt_ values based on geographic coordinates [26].

Within this modeling framework, the GLMM accounts for both deterministic effects of environmental covariates and spatially structured variability. A simplified representation of the model is given in Equation (5):

(5) $$\delta^{18}O\;(S_{i})\;=\;\beta_{0}+\beta_{1}.DEM+\beta_{2}.T+\mu (S_{i})$$

where δ^18^O(S_i_) is the isotopic value at location S_i_, *ꞵ*_0_ is the intercept, *ꞵ*_1_ and *ꞵ*_2_ are regression coefficients associated with elevation and temperature, respectively, and μ(S_i_) represents the spatially correlated residual term, typically modeled using a Matèr covariance structure [27,28].

All environmental predictors were subsequently extracted for each sampling site based on their geographic coordinates (Table 1). In addition, interpolated *δ*^18^O_ppt_ values derived from the IsoriX model [7] were assigned to each location and used as explanatory variables in subsequent modeling of δ^18^O in wood cellulose [29]. The resulting spatial patterns in *δ*^18^O_ppt_ reflect large-scale atmospheric processes, including Rayleigh distillation and temperature-dependent fractionation along air-mass transport pathway [30,31].

### 4.3. Construction of the Isotopic Model

#### Multiple Linear Regression Model

The isotopic modeling framework was implemented in R using the caret package [32,33,34]. Prior to model fitting, all predictor variables were standardized by centering (subtracting the mean) and scaling (dividing by the standard deviation), ensuring comparability among predictors and facilitating interpretation of their relative contributions.

A full model was initially specified (Equation (6)), incorporating a comprehensive set of environmental and climatic variables hypothesized to influence *δ*^18^O variability across the Amazon region:

(6) $$\delta^{18}O\;=\;\beta_{0}+\beta_{1}VPD+\beta_{2}RH+\beta_{3}PET+\beta_{4}DEM+\beta_{5}PA+\beta_{6}MAT+\beta_{7}MAP+\beta_{8}\delta^{18}O$$

Model selection was performed using an exhaustive approach, in which all possible subsets of predictor variables were evaluated through the *dredge* function in. This procedure generated a total of 256 candidate models. Model ranking followed an information-theoretic framework, with selection based on Akaike’s Information Criterion corrected for small sample sizes (AICc). Models with ΔAICc < 4 were considered to have substantial support [35,36], resulting in a reduced set of five competing models (Table 3).

Model performance was assessed using Leave-One-Out-Cross-Validation (LOOCV) [37], a resampling strategy in which each observation is iteratively excluded from model calibration and used for independent validation. Predictive performance was quantified using the coefficients of determination (R^2^), root mean square error (RMSE), and mean absolute error (MAE). The final model was then applied to gridded environmental predictors to generate continuous spatial predictions (isoscapes), prioritizing the most parsimonious model.

To explore model robustness and its relevance for forensic applications, we conducted parallel analyses using both individual tree *δ*^18^O measurements and site-level averages. This comparison addresses a key constraint in timber provenance studies: while averaging multiple samples reduces local variability and improves statistical performance, forensic investigations typically rely on single-sample analyses. Consequently, models based on individual observations provide a more realistic framework for real-world applications, despite inherent greater uncertainty associated with within-site variability. This trade-off highlights the balance between statistical accuracy and practical applicability in forensic contexts.

Finally, spatial autocorrelation in model residual was evaluated using Moran’s I statistic. The analysis indicated no significant spatial structure (*p* = 0.73), suggesting that residuals were spatially independent and supporting the decision not to incorporate geostatistical interpolation methods such as kriging.

### 4.4. Random Forest

Random Forest (RF) is a machine learning (ML) technique applied in modeling [38]. We predicted δ^18^O of cellulose using RF and tested different covariates to select the most accurate model. Recursive feature elimination (RFE) was applied to select representative variables using the caret package. RFE constructs a model with all variables (Table 4), ranks their importance, and iteratively eliminates the least important. Model performance was evaluated using repeated 5-fold cross-validation (10 repetitions) via the trainControl function. Metrics such as RMSE and R^2^ were obtained from this process.

Like for the multiple regression model, we trained Random Forest models using individual tree-level *δ*^18^O values and site-averaged *δ*^18^O values to evaluate model performance and applicability to timber provenance. For provenance applications specifically, the model trained on site-mean values was selected for isoscape prediction and geographic assignment due to several critical methodological considerations. However, we also trained a Random Forest model using individual tree-level *δ*^18^O values in order to evaluate the full isotopic variability, including within-site variation. This allowed us to assess how the inclusion of biological noise arising from factors such as species differences, microclimatic conditions, and tree age impacts model performance.

The RF model was trained using caret package, with a grid search for tuning hyperparameters (Table 2). Variable importance was assessed, and predictions were compared with the values observed. Environmental rasters were used to predict *δ*^18^O values across the Amazon Basin, and an isoscape was created and plotted using ggplot2 package [38], with the Legal Amazon boundaries serving as a reference. To better understand the structure of isotopic variability, we trained two Random Forest models: one using individual *δ*^18^O values from each tree and another using site-level means.

## 5. Conclusions

This study provides the first regional *δ*^18^O isoscapes for Amazonian wood and offers important methodological insights for isotope-based timber provenance in tropical forests. Although spatial autocorrelation was not statistically detected in our dataset, the coherent east–west gradients observed in the predictive maps indicate that isotopic structure likely exists but was not fully captured at the current sampling scale. Both multiple linear regression and Random Forest models generated broadly similar spatial patterns and comparable predictive errors; however, differences in predicted isotopic range were more evident in regions undergoing rapid environmental transformation, such as the arc of deforestation, highlighting that model selection may influence spatial interpretation and assignment sensitivity. By explicitly comparing site-averaged and individual-tree approaches, we demonstrate a fundamental trade-off between statistical robustness and forensic realism: while site means improve model fit and correlation strength, individual-level data more accurately reflect real-world enforcement scenarios, despite greater within-site variability. These findings underscore that isoscape development in highly heterogeneous tropical forests must balance generalizability with operational applicability. Future advances will depend on expanding spatial sampling coverage and integrating ecophysiologically relevant covariates, including traits such as stomatal conductance, successional status, and wood density, which influence plant water use and isotopic discrimination processes. In this sense, Amazonian timber isoscapes should no longer be regarded as static climatic surfaces, but as dynamic eco-physiological representations of forest structure, function, and environmental change.

## Figures and Tables

**Table 1:** Average data of environmental variables extracted from WorldClim (2020).

Site	State	Lat DD	Lon DD	VPD Kpa	RH%	PET mm	DEM m	MAT °C	MAP mm	δ^18^Oppt ‰
Maués	Amazonas	−57.589	−3.996	0.95	72.16	1390	51	27.00	2209.36	−3.81
São Gabriel da Cachoeira	Amazonas	−67.013	−0.121	0.90	72.45	1256	97	25.97	2902.27	−3.93
Rurópolis	Pará	−54.908	−3.992	1.09	67.93	1320	120	25.94	1830.03	−3.55
Juruá	Amazonas	−66.055	−3.907	0.93	71.87	1235	81	26.45	2849.93	−4.57
Atalaia do Norte	Amazonas	−70.291	−4.304	0.89	72.81	1205	107	26.45	2702.18	−5.12
Manicoré	Amazonas	−61.869	−6.010	0.97	71.57	1276	64	26.84	2751.36	−4.45
ZF2	Amazonas	−60.150	−2.638	1.08	68.02	1328	97	26.39	2228.53	−4.06
Itapiranga	Amazonas	−59.121	−2.493	0.96	70.90	1361	113	26.47	2274.51	−3.99
Tanguro	Mato Grosso	−52.377	−13.081	1.26	60.52	1521	381	24.76	1621.02	−3.71
Itapiranga	Amazonas	−59.121	−2.497	0.96	70.90	1361	113	26.47	2274.51	−3.99
Belterra	Pará	−54.974	−3.398	1.09	67.64	1313	144	25.73	1847.88	−3.54
Rurópolis	Pará	−54.908	−3.992	1.09	67.93	1320	120	25.94	1830.03	−3.55
Uruará	Pará	−53.871	−2.974	1.08	67.43	1309	196	25.27	1897.40	−3.31
Barcelos	Amazonas	−62.382	−1.764	0.91	72.65	1299	34	26.38	2576.45	−4.03
Pauini	Amazonas	−69.036	−8.484	0.99	69.01	1182	207	24.62	2352.68	−5.39
Itapuã do Oeste	Rondônia	−63.083	−9.366	1.10	67.08	1257	138	25.23	2249.53	−4.91
Barra do Bugres	Mato Grosso	−57.993	−14.906	1.24	61.26	1481	282	24.79	1695.15	−4.72
Santa Maria das Barreiras	Pará	−50.429	−8.709	1.31	61.42	1467	216	25.83	1744.29	−3.06
Ferreira Gomes	Amapá	−51.909	1.100	1.07	67.06	1530	248	25.48	2506.87	−2.84
Cáceres	Mato Grosso	−58.253	−16.264	1.29	60.20	1623	233	26.11	1378.89	−4.66
Porto Esperidião	Mato Grosso	−58.697	−15.882	1.38	58.47	1640	198	26.24	1466.49	−4.70
Caracaraí	Roraima	−61.088	1.604	1.08	68.66	1506	60	27.33	1809.22	−3.84
Silves	Amazonas	−58.569	−2.773	1.09	67.82	1365	77	26.74	2190.13	−3.90
Parauapebas	Pará	−50.560	−5.805	1.18	63.98	1383	233	25.22	1898.79	−3.04
Bragança	Pará	−46.681	−0.940	1.16	66.21	1562	14	26.34	2369.48	−1.85

*Lat DD* Latitude in decimal degrees, *Lon DD* Longitude in decimal degrees, *VPD* Vapor Pressure Deficit, *RH* Relative Humidity, *PET* Potential Evapotranspiration, *DEM* Digital Elevation Model, *MAT* Mean Annual Temperature, *MAP* Mean Annual Precipitation, δ^18^Oppt δ^18^O of Precipitation.

**Table 2:** Quality metrics for multiple linear regression (MLR) and Random Forest (RF) models used to predict the *δ*^18^O of tree-ring cellulose in the Amazon region. Models were trained using either individual tree-level data or site-averaged isotopic values. MAE is the mean absolute error; RMSE is the root mean square error; R^2^ is the coefficient of determination. For the MLR models, metrics were calculated from observed versus predicted values. For the RF models, metrics were estimated using internal leave-one-out cross-validation via the caret package in R.

	MLR-Individual	MLR-Site Average	RF-Individual	RF-Site Average
MAE	0.92	0.56	0.90	0.64
RMSE	1.10	0.68	1.12	0.77
R^2^	0.42	0.70	0.44	0.67
Std.dev (‰)				
Min.	1.15	0.72	0.01	0.17
1st Q	1.16	0.75	0.95	0.70
Median	1.17	0.78	1.25	0.87
3rd Q	1.19	0.83	1.48	1.10
Max.	2.00	1.86	1.97	2.00

**Table 3:** Models selected by information theory, ΔAICc < 4.

	Models	ΔAICc < 4
1	$\delta^{18}O=\beta_{1}DEM+\beta_{2}Isorix+\beta_{3}MAP+\beta_{4}MAT+\beta_{5}PET+\beta_{6}RH+\beta_{7}VDP$	4456
2	$\delta^{18}O=\beta_{1}DEM+\beta_{2}Isorix+\beta_{3}MAP+\beta_{4}MAT+\beta_{5}RH+\beta_{6}VPD$	4458
3	$\delta^{18}O=\beta_{1}DEM+\beta_{2}Isorix+\beta_{3}MAP+\beta_{4}PET+\beta_{5}RH+\beta_{6}VPD$	4459

**Table 4:** Hyperparameter for variable selection in the Random Forest model.

Hyperparameter	Description	Unit
Longitude	Geographic coordinate (X)	Decimal degrees (°)
Latitude	Geographic coordinate (Y)	Decimal degrees (°)
VPD	Vapor pressure deficit	KPa
RH	Relative humidity	%
PET	Potential evapotranspiration	mm
DEM	Digital elevation model	Meters (m)
MAT	Annual average temperature	°C
MAP	Annual average precipitation	mm
*δ*^18^O*_ppt_*	Predictive model of *δ*^18^O in precipitation	‰
*δ*^18^O	Isotopic ratio of ^18^O	‰

## Data Availability

Data will be made available on request.

## Supplementary Materials

The following supporting information can be downloaded at: https://www.mdpi.com/article/10.3390/molecules31091542/s1.
