# Supplementary material for: Isoscape of Oxygen Stable Isotopes in Woods of the Amazon
Source: Molecules. 2026 May 6;31(9):1542. doi: 10.3390/molecules31091542 (PMC13165382; doi:10.3390/molecules31091542)
Supplement: Supplementary file 1 [file molecules-31-01542-s001.zip › molecules-4211466-supplementary.pdf]

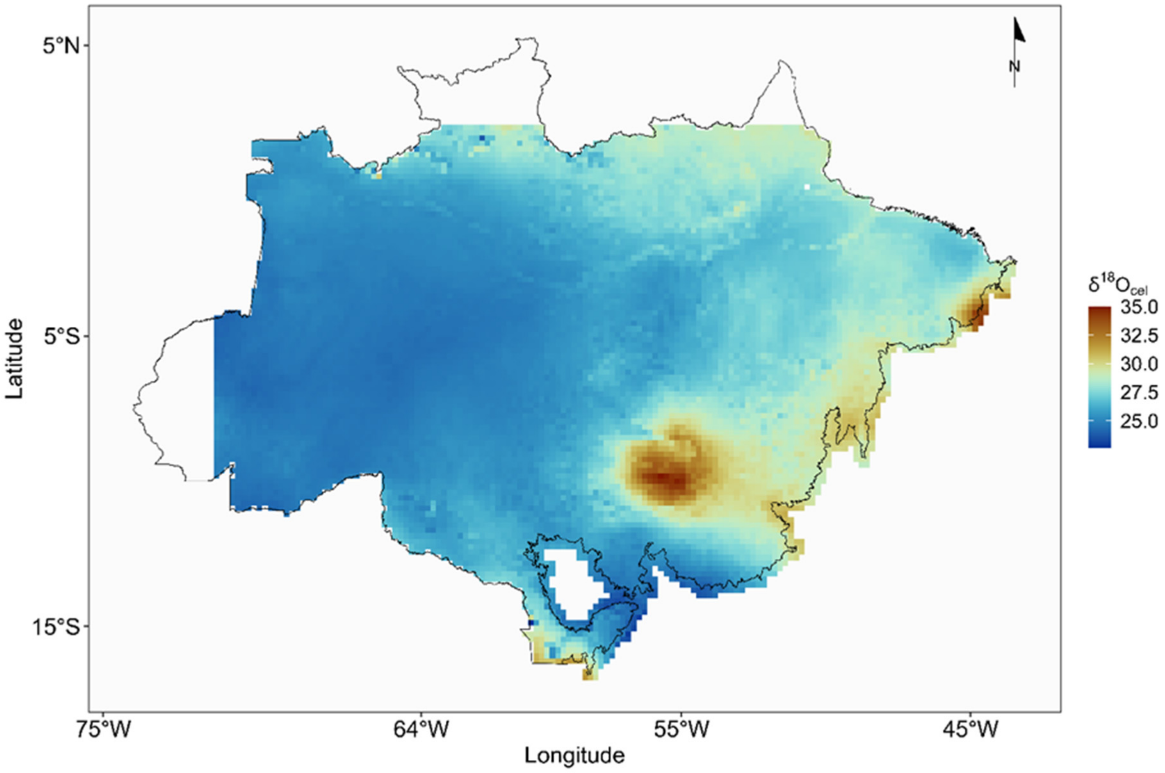

Figure S1. Average isoscapes of $\delta^{18}O$ in cellulose across the Amazon region, modeled using multiple linear regression (MLR) based on individual trees $\delta^{18}O$ values. Black lines indicate the boundaries of the Amazon region. Warmer colors represent higher $\delta^{18}O$ values, while cooler colors indicate lower values.

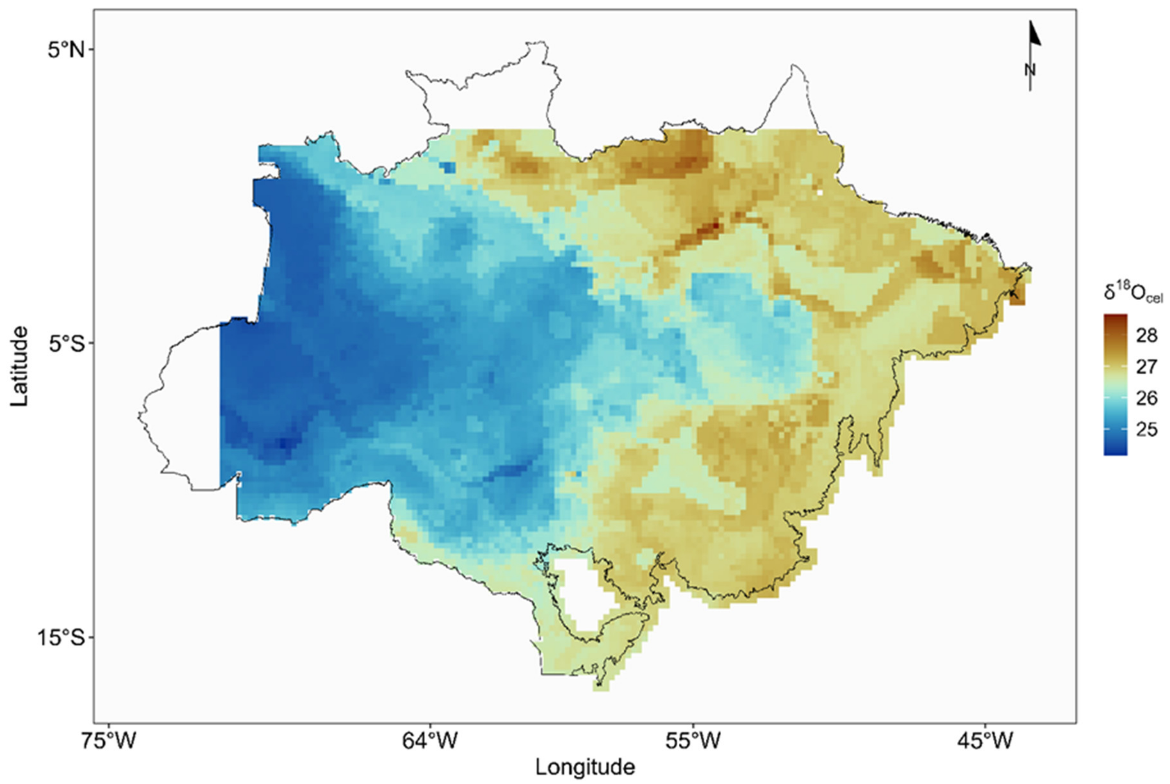

Figure S2. Average isoscapes of $\delta^{18}O$ in cellulose across the Amazon region, modeled using random forest model (RF) based on individual trees $\delta^{18}O$ values. Black lines indicate the boundaries of the Amazon region. Warmer colors represent higher $\delta^{18}O$ values, while cooler colors indicate lower values.

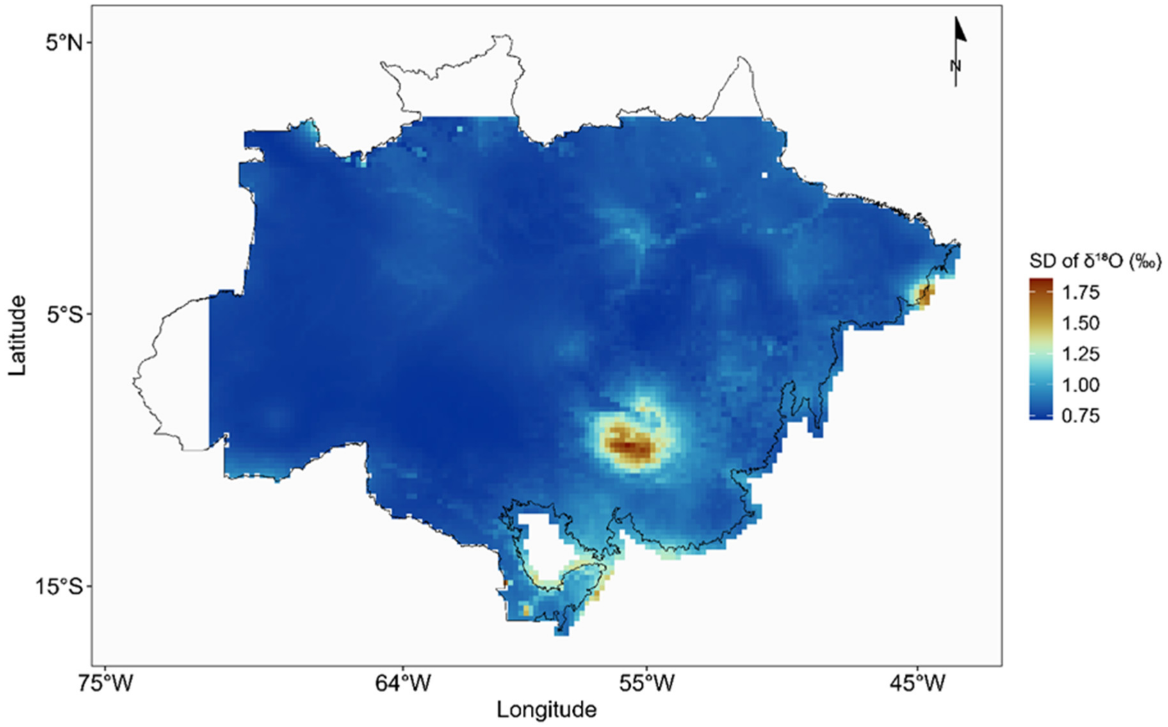

Figure S3. Standard deviation isoscapes of $\delta^{18}O$ in cellulose across the Amazon region, modeled using multiple linear regression (MLR) based on site-average $\delta^{18}O$ values. Black lines indicate the boundaries of the Amazon region. Warmer colors represent higher $\delta^{18}O$ values, while cooler colors indicate lower values.

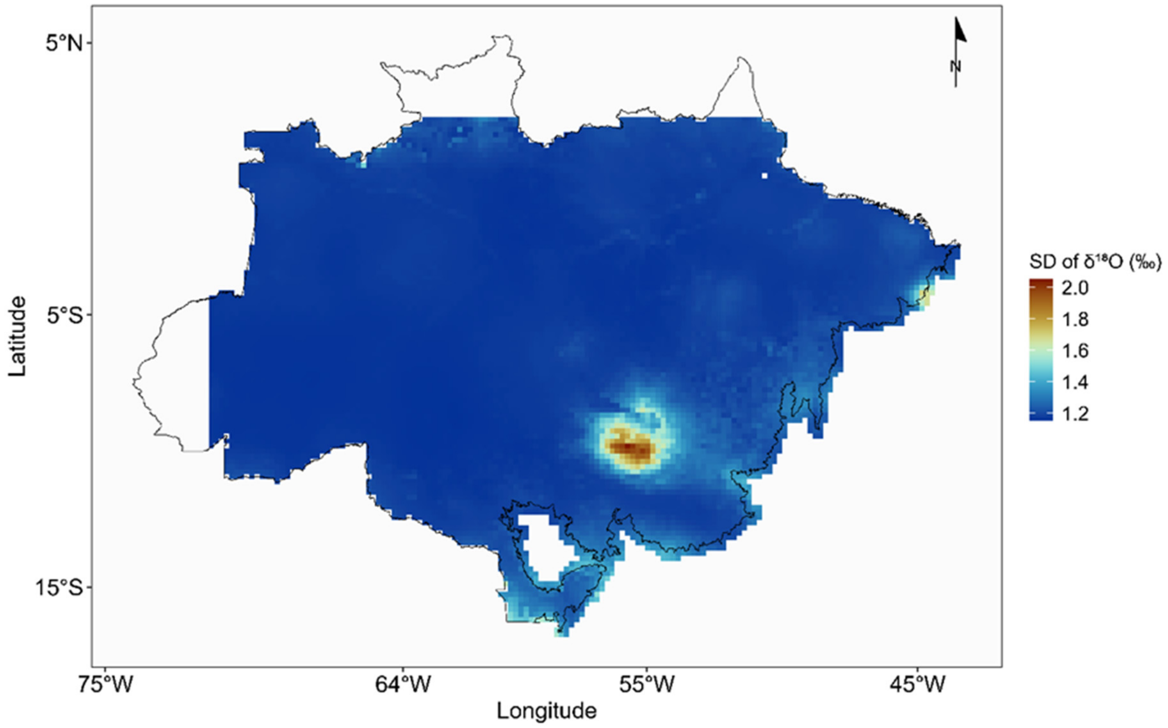

Figure S4. Standard deviation isoscapes of $\delta^{18}O$ in cellulose across the Amazon region, modeled using multiple linear regression (MLR) based on individual trees $\delta^{18}O$ values. Black lines indicate the boundaries of the Amazon region. Warmer colors represent higher $\delta^{18}O$ values, while cooler colors indicate lower values.

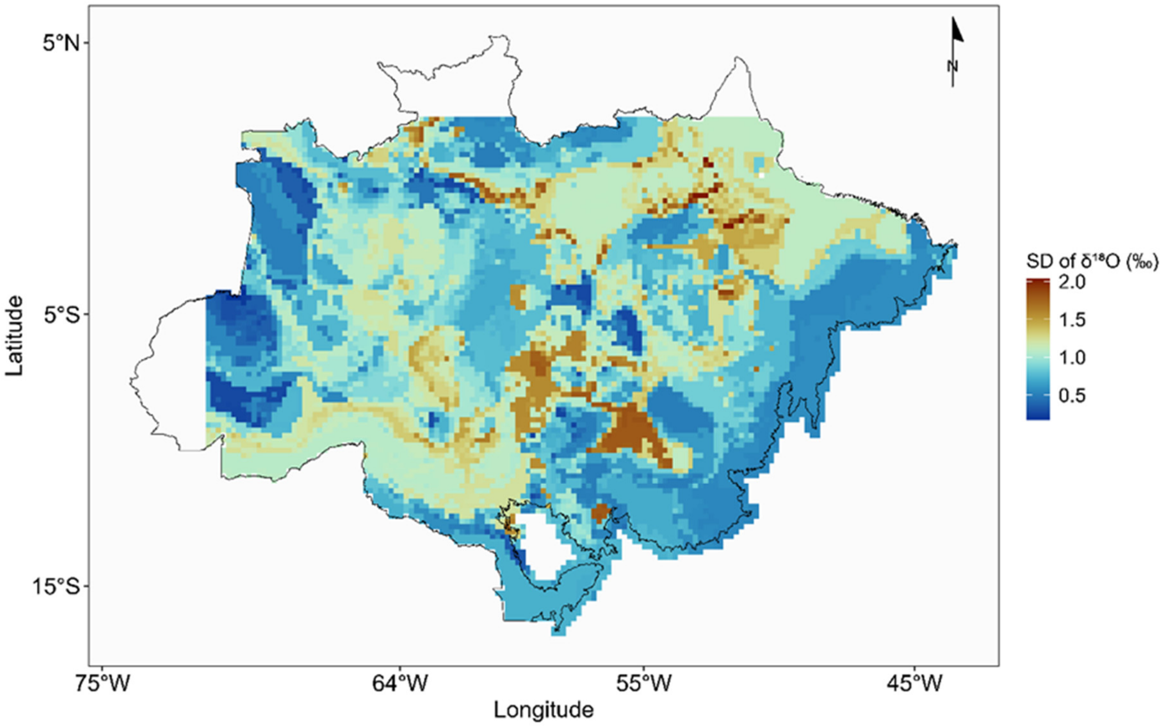

Figure S5. Standard deviation isoscapes of $\delta^{18}O$ in cellulose across the Amazon region, modeled using random forest model (RF) based on site-average $\delta^{18}O$ values. Black lines indicate the boundaries of the Amazon region. Warmer colors represent higher $\delta^{18}O$ values, while cooler colors indicate lower values.

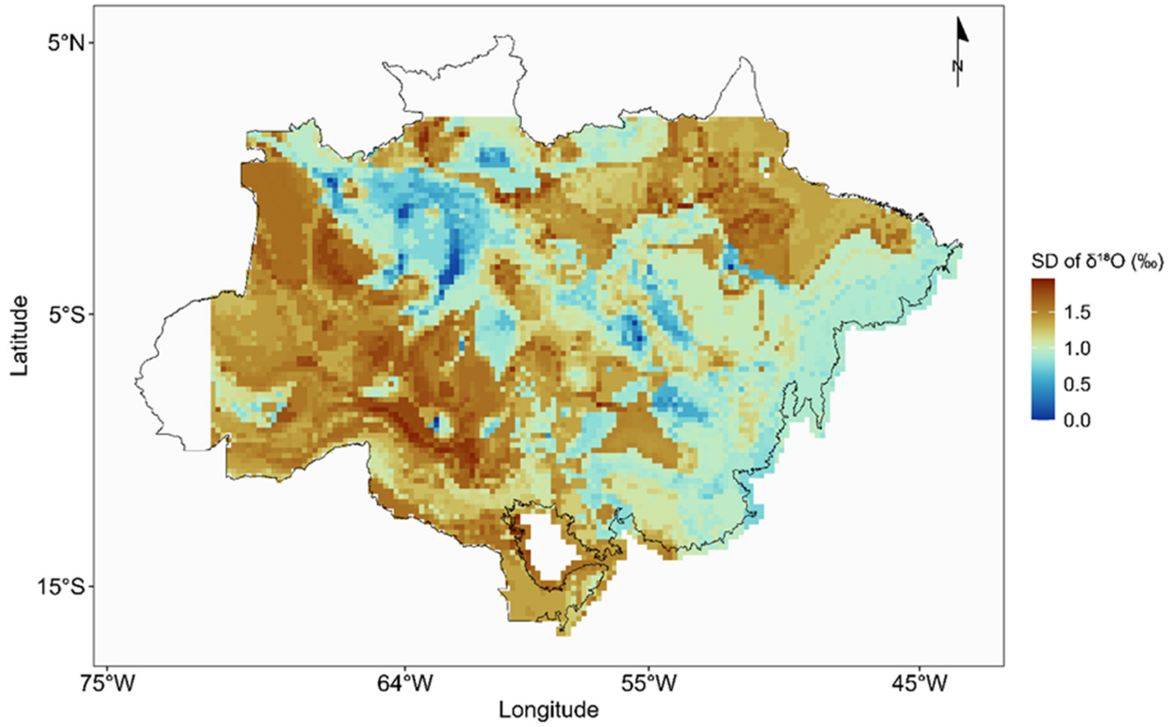

Figure S6. Standard deviation isoscapes of $\delta^{18}O$ in cellulose across the Amazon region, modeled using random forest model (RF) based on individual tree $\delta^{18}O$ values. Black lines indicate the boundaries of the Amazon region. Warmer colors represent higher $\delta^{18}O$ values, while cooler colors indicate lower values.

Table S1. Ecological characteristics and vegetation types of the species collected during the project. The table presents the ecological classification of species according to their successional group (pioneer, early secondary, late secondary, or climax), as well as the vegetation types in which they occur, including primary and secondary forests, dense ombrophilous forests, semi-deciduous seasonal forests, non-flooded forests, várzea, and igapó.

Species	Genus	Family	Ecological group	Sociological importance
Amburana sp.	*Amburana*	Fabaceae	Shade-tolerant climax	Non-flooded forest
Cedrela fissilis	*Cedrela*	Meliaceae	Late-successional, climax, or light-demanding climax	Dense ombrophilous forest
Dipteryx odorata	*Dipteryx*	Fabaceae	Light-demanding climax	Dense ombrophilous, non-flooded, and high várzea forest
Handronathus sp.	*Handroanthus*	Bignoniaceae	Early or late-successional, light-demanding climax	Dense ombrophilous, and non-flooded forest
Myroxylon balsamum	*Myroxylon*	Fabaceae	Pioneer	Non-flooded forest
Protium sp.	*Protium*	Burseraceae	Early successional	Non-flooded forest
Iryanthera macrophylla	*Iryanthera*	Myristicaceae	Pioneer	Non-flooded forest
Hymenolobium modestum	*Hymenolobium*	Fabaceae	Light-demanding pioneer	Non-flooded forest
Dinizia excelsa	*Dinizia*	Fabaceae	Light-demanding climax	Dense ombrophilous, and non-flooded forest
Clarisia racemosa	*Clarisia*	Meliaceae	Late successional	Dense ombrophilous forest
Hymenaea courbaril	*Hymenaea*	Fabaceae	Late-successional or light-demanding climax	Dense ombrophilous forest
Ocotea rubra Mez	*Ocotea*	Lauraceae	Late successional	Dense ombrophilous, and non-flooded forest
Mezilaurus itauba	*Mezilaurus*	Lauraceae	Late successional	Dense ombrophilous, and non-flooded forest
Manilkara huberi	*Manilkara*	Sapotaceae	Late successional or climax	Dense ombrophilous, and non-flooded forest
Qualea paraensis	*Qualea*	Vochysiaceae	Late successional	Non-flooded forest
Aspidosperma desmanthum	*Aspidosperma*	Apocynaceae	Late successional	Non-flooded forest
Qualea sp.	*Qualea*	Vochysiaceae	Late successional	Non-flooded forest
Protium subserratum	*Protium*	Burseraceae	Late successional	Dense ombrophilous, and non-flooded forest
Protium hebetatum	*Protium*	Burseraceae	Late successional	Dense ombrophilous, and non-flooded forest
Cedrela odorata	*Cedrela*	Meliaceae	Late-successional or light-demanding climax	Dense ombrophilous forest
Anadenanthera sp.	*Anadenanthera*	Fabaceae	Pioneer	Non-flooded forest
Anacardium sp.	*Anacardium*	Anacardiaceae	Late successional	Non-flooded forest
Scleronema micranthum	*Scleronema*	Malvaceae	Late successional	Non-flooded forest
Eschweilera coriacea	*Eschweilera*	Lecythidaceae	Light-demanding climax	Non-flooded forest
Brosiumum rubescens	*Brosimum*	Moraceae	Pioneer and late-successional	Non-flooded forest

Micrandropsis scleroxylon	*Micrandropsis*	Fabaceae	Late successional	Campinarana, non-flooded forest
Erisma lanceolatum	*Erisma*	Vochysiaceae	Climax	Non-flooded forest
Goupia glabra	*Goupia*	Euphorbiaceae	Climax	Non-flooded forest
Trattinnickia rhoifolia	*Trattinnickia*	Burseraceae	Shade-tolerant pioneer	Primary forest non-flooded
Trattinnickia burseraefolia	*Trattinnickia*	Burseraceae	Shade-tolerant pioneer	Primary forest non-flooded
Trattinnickia glaziovii	*Trattinnickia*	Burseraceae	Shade-tolerant pioneer	Primary forest non-flooded
Aspidosperma excelsum	*Aspidosperma*	Apocynaceae	Early or late successional	Non-flooded forest
Terminalia tetraphylla	*Terminalia*	Combretaceae	Late successional	Dense ombrophilous forest
Mouriri brachyanthera	*Mouriri*	Melastomataceae	Late successional	Dense ombrophilous forest
Ormosia paraensis	*Ormosia*	Fabaceae	Climax	Non-flooded forest
Virola sebiafera	*Virola*	Myristicaceae	Pioneer	Secondary forest
Hymenaea sp.	*Hymenaea*	Fabaceae	Late-successional or light-demanding climax	Seasonal semi-deciduous, and ombrophilous florest
Swartzia sp.	*Swartzia*	Fabaceae	Late successional	Várzea and igapó forest
Bertholletia excelsa	*Berttholletia*	Lecythidaceae	Climax	Dense ombrophilous, and non-flooded forest
Pouteria guianensis	*Pouteria*	Sapotaceae	Climax	Igapó, várzea, seasonal evergreen, seasonal semi-deciduous and ombrophilous forest
Cedrelinga catenaeformis	*Cedrelinga*	Fabaceae	Late successional or climax	Seasonal evergreen and non-flooded forest
Couratari stellata	*Couratari*	Lecythidaceae	Climax	Igapó, non-flooded and ombrophilous forest
Peltogyne paniculata	*Peltogyne*	Fabaceae	Late successional or climax	Non-flooded, ombrophilous forest and Amazonian savanna
Nectandra sp.	*Nectandra*	Lauraceae	Late successional	Ombrophilous and mixed ombrophilous forest
Caryocar sp.	*Caryocar*	Caryocaraceae	Pioneer	Dense ombrophilous forest
Bowdichia sp.	*Bowdichia*	Fabaceae	Pioneer, later successional and climax	Dense ombrophilous, and non-flooded forest
